# Astrocytic Neuroligins Are Not Required for Synapse Formation or a Normal Astrocyte Cytoarchitecture

**DOI:** 10.1101/2023.04.10.536254

**Published:** 2023-04-10

**Authors:** Samantha R. Golf, Justin H. Trotter, George Nakahara, Thomas C. Südhof

**Affiliations:** 1 Dept. of Molecular and Cellular Physiology, Stanford University School of Medicine, Stanford, CA 94305, USA.; 2 Howard Hughes Medical Institute, Stanford University School of Medicine, Stanford, CA 94305, USA.

## Abstract

Astrocytes exert multifarious roles in the formation, regulation, and function of synapses in the brain, but the mechanisms involved remain unclear. Interestingly, astrocytes abundantly express neuroligins, postsynaptic adhesion molecules that bind to presynaptic neurexins. A pioneering recent study reported that loss-of-function of neuroligins in astrocytes impairs excitatory synapse formation and astrocyte morphogenesis. This study suggested a crucial synaptic function for astrocytic neuroligins but was puzzling given that constitutive neuroligin deletions do not decrease excitatory synapse numbers. Thus, we here examined the function of astrocytic neuroligins using a rigorous conditional genetic approach with deletion of all major neuroligins (*Nlgn1–3*) in astrocytes. Our results show that early postnatal deletion of neuroligins from astrocytes has no effect on cortical or hippocampal synapses and does not alter the cytoarchitecture of astrocytes. Thus, astrocytic neuroligins are unlikely to shape synapse formation or astrocyte development but may perform other important functions in astrocytes.

## INTRODUCTION

**Figure 1:**
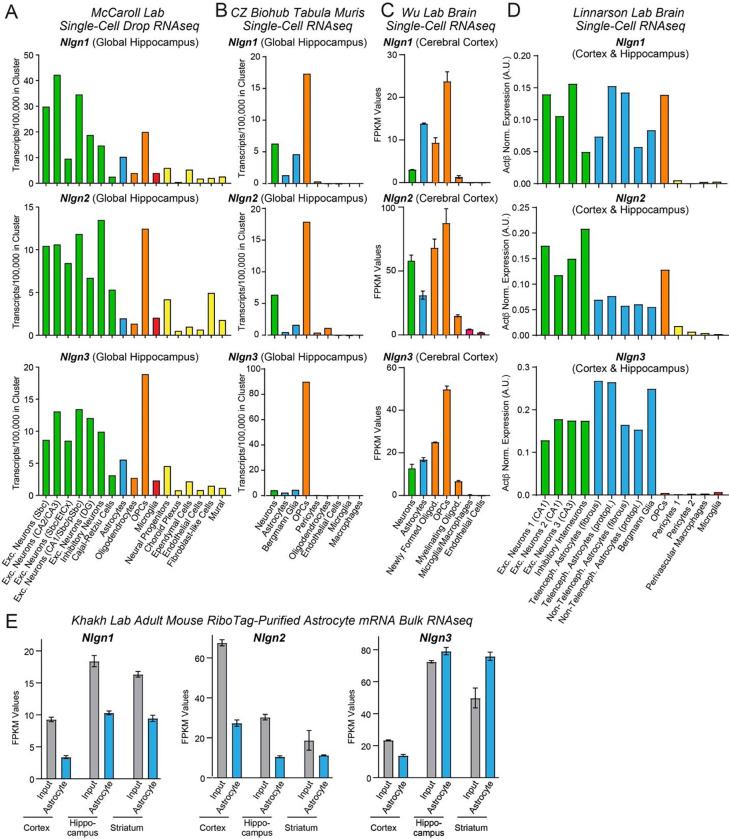
Neuroligin genes (*Nlgn1*-*3*) are abundantly expressed by neurons, astrocytes, and OPCs in brain as determined by analyses of publicly available RNAseq datasets (**A-C**) Analysis of *Nlgn1*, *Nlgn2*, and *Nlgn3* mRNA levels in neurons (green), astrocytes (blue), oligodendrocyte lineage cells (orange), microglia (red), and other cell types in the brain (yellow) using the single-cell RNAseq dataset published from the McCaroll lab (Saunders and Macosko et al., 2018, www.dropviz.org) (**A**), Chan Zuckerberg Initiative (Schaum et al., 2018) (**B**), Wu lab (Zhang et al., 2014) (**C**), and Linnarson lab (Zeisel et al., 2018, www.mousebrain.org) (**D**). Note that although relative expression levels vary greatly between datasets, all datasets support the conclusion that Nlgn1, Nlgn2, and Nlgn3 are broadly expressed in astrocytes and OPCs. (**E**) Analysis of *Nlgn1*, *Nlgn2*, and *Nlgn3* mRNA levels in astrocytes in three different brain regions (cortex, hippocampus, and striatum) using the bulk RNAseq datasets published by the Khakh lab (Chai et al., 2017; Srinivasan et al., 2016, www.astrocyternaseq.org) that examined RiboTag-purified mRNAs. Note that *Nlgn4* is not measured in the RNAseq experiments shown, probably because its expression levels are low and because its mRNA is very GC rich. For an analysis based on the pioneering original Barres lab data that used a less deep sequencing approach, see Figure S1.

Astrocytes play a vital role across the lifespan of a synapse, with important functions at every point from synapse formation to synaptic transmission to synapse elimination (reviewed in Farhy-Tselnicker and Allen, 2018; Eroglu and Barres 2010; Shan et al., 2021; Verkhratsky and Nedergaard, 2018; Lyon and Allen, 2022; Nagai et al., 2021). The emergence of novel tools to genetically access and interrogate astrocytes has paved the way for a new field dedicated to understanding the molecular mechanisms by which astrocytes influence synapses (Yu et al., 2020). Moreover, recent RNA sequencing (RNAseq) studies have revealed astrocytic expression of cell-adhesion molecules that were traditionally viewed as specifically *synaptic* in nature. The astrocytic expression of these synaptic cell-adhesion molecules gives rise to a parsimonious and attractive hypothesis that accounts for how astrocytes form interactions with synapses at the molecular level (Saint-Martin and Goda, 2022; Tan and Eroglu, 2021). This hypothesis posits that astrocytes are integrated into tripartite synapses via these adhesion molecules and that positive or negative signaling mediated by these adhesion molecules regulates synapse formation, synaptic function, and synapse elimination. Prominent among synaptic cell-adhesion molecules that are expressed by astrocytes are neuroligins, a family of postsynaptic adhesion molecules that bind to presynaptic neurexins, presynaptic LAR-type receptor tyrosine phosphatases, and postsynaptic MDGA proteins (Liu et al., 2022; Liu et al., 2023; Connor et al., 2019; Qin et al., 2020; Südhof 2008; Bemben et al., 2015; Yoshida et al., 2021) (see below, Figure 1).

Mice express four neuroligin genes (*Nlgn1–4*) that encode homologous proteins with an identical domain structure and a high degree of sequence similarity (Ichtchenko et al., 1995 and 1996; Bolliger et al. 2008). Despite their similarity, however, neuroligins display different localizations in brain and perform distinct non-redundant functions (Zhang et al., 2015; Chanda et al., 2018). Specifically, *Nlgn1* protein is found only at glutamatergic excitatory synapses (Song et al., 1999), *Nlgn2* at GABAergic inhibitory and cholinergic synapses (Graf et al., 2004; Varoqueaux et al., 2004; Panzanelli et al., 2017; Hoon et al., 2009; Patrizi et al., 2008; Takacs et al., 2013 and 2018), and *Nlgn3* at both (Budreck and Scheiffele, 2007). *Nlgn4*, conversely, is present in glycinergic synapses in mice (Hoon et al., 2011; Zhang et al., 2018) but is expressed only at lower levels in brain and in human neurons functions at excitatory synapses (Marro et al., 2019), making a definitive assignment of *Nlgn4* to synapse types difficult.

Since astrocytes physically interact with synapses and carry out important functions at synapses, astrocytic neuroligins are ideally positioned to control synapses, which neuroligins could do via binding to other cell-adhesion molecules and regulating astrocyte-neuron interactions at tripartite synapses. Indeed, a landmark study showed that astrocytic expression of neuroligins controls synapse formation in mice and that one specific neuroligin isoform in particular, *Nlgn2*, plays a central role in enabling excitatory synapse formation in the visual cortex (Stogsdill *et al*. 2017). Moreover, Stogsdill et al. (2017) revealed that *Nlgn2* and other neuroligins control the cytoarchitecture of astrocytes since a loss-of-function of *Nlgn2* and other neuroligins greatly reduced astrocytic branching in mixed cortical cultures of glia and neurons and in the visual cortex in vivo, leading even to a complete loss of astrocyte arborization. Although the dramatic results by Stogsdill et al. (2017) have guided all recent reviews on tripartite synapses, they were puzzling because *Nlgn2* protein has only been detected in inhibitory but not in excitatory synapses by immunocytochemistry (Graf et al., 2004; Varoqueaux et al., 2004; Panzanelli et al., 2017; Hoon et al., 2009; Patrizi et al., 2008; Takacs et al., 2013 and 2018). It was thus difficult to understand how astrocytic *Nlgn2* could control excitatory synapses since *Nlgn2* appears to be absent from excitatory synapses. Moreover, deletions of *Nlgn2* that remove the protein from both neurons and glia cells did not alter the number of strength of excitatory synapses but severely impaired inhibitory synapses (Varoqueaux et al., 2006; Chubykin et al., 2007). In addition, the constitutive knockout (KO) of Nlgn1–3 had no effect on excitatory or inhibitory synapse numbers (Varoqueaux et al. 2006). Viewed together, these results are difficult to reconcile with each other, prompting fundamental questions about the role of astrocytic neuroligins in synapses.

The importance of understanding the potential role of astrocytic neuroligins in synapse formation, the high level of neuroligin expression in astrocytes, and the discrepancies between previous results motivated us in the present study to examine the fundamental functions of astrocytic neuroligins. Using a rigorous genetic approach, we show that early postnatal deletions of *Nlgn1–3* selectively in astrocytes does not significantly alter excitatory or inhibitory synapse numbers or functions (*Nlgn4* was not targeted owing to its low expression levels). Moreover, *Nlgn1–3* deletions had no effect on the cytoarchitecture of astrocytes. Thus, astrocytic neuroligins likely do not function to shape the morphology of astrocytes or the contribution of astrocytes to tripartite synapses but probably have other important non-synaptic roles.

## RESULTS

### Astrocytes abundantly express *Nlgn1–3*

For a gene to be involved in a particular physiological process, it must be expressed in the right place at the right time. To assess the expression of neuroligins in astrocytes in comparison with other cell types in brain, we examined the mRNA levels of *Nlgn1–3* in various brain cells by analysis of published RNA sequencing (RNAseq) datasets (Figure 1, S1). *Nlgn4* mRNAs were not detected in these datasets, possibly because of the low expression levels or high GC content of *Nlgn4* mRNAs. We quantified *Nlgn1*, *Nlgn2*, and *Nlgn3* mRNA transcripts in astrocytes and other cell types in the mouse hippocampus and cortex in the single-cell RNAseq datasets from the McCaroll Lab (Saunders and Macosko et al., 2018, www.dropviz.org), Chan Zuckerberg Initiative (Schaum et al. 2018), Wu lab (Zhang et al. 2014), and Linnarson lab (Zeisel et al., 2018, www.mousebrain.org) (Figure 1A–D). Interestingly, these quantifications showed that all three neuroligins were abundantly expressed in astrocytes and oligodendrocyte precursor cells (OPCs) in addition to neurons (Figure 1A–D). The relative expression levels of *Nlgn1*, *Nlgn2*, and *Nlgn3* in different cell types differed, probably because different RNAseq procedures and data analysis algorithms were used, but all datasets revealed similarly high neuroligin expression levels in neurons and astrocytes and generally even higher expression levels in OPCs. *Nlgn3* was consistently the most astrocyte-enriched neuroligin isoform (Figure 1A). A prominent expression of neuroligins in astrocytes was further supported by bulk RNAseq experiments performed by the Khakh lab (Chai et al., 2017; Srinivasan et al., 2016, www.astrocyternaseq.org) using astrocytic mRNAs purified via the RiboTag approach (Heiman et al., 2008) from the cortex, hippocampus, or striatum (Figure 1E). Again, all three isoforms were detected across these brain regions, with *Nlgn3* identified as the most enriched isoform compared to the input.

In mice, the major period for developmental synaptogenesis occurs over the first three weeks of life (Semple et al., 2013), although synapses are continuously eliminated and reformed throughout life. Measurements of the expression of neuroligins in brain as a function of postnatal development showed that neuroligin expression parallels the process of synaptogenesis (Irie et al., 1997). Moreover, comparative analysis of RNAseq data from the Barres lab (Clarke et al., 2018, www.brainrnaseq.org) indicates that during this period, astrocytes in the cortex, hippocampus, and striatum also express *Nlgn1–3* (Figure S1), consistent with a role in the establishment of neural circuits. Thus, neuroligins are expressed in the right place and time for a role as astrocytic drivers of synapse formation.

### Conditional deletion of *Nlgn1–3* in astrocytes

**Figure 2:**
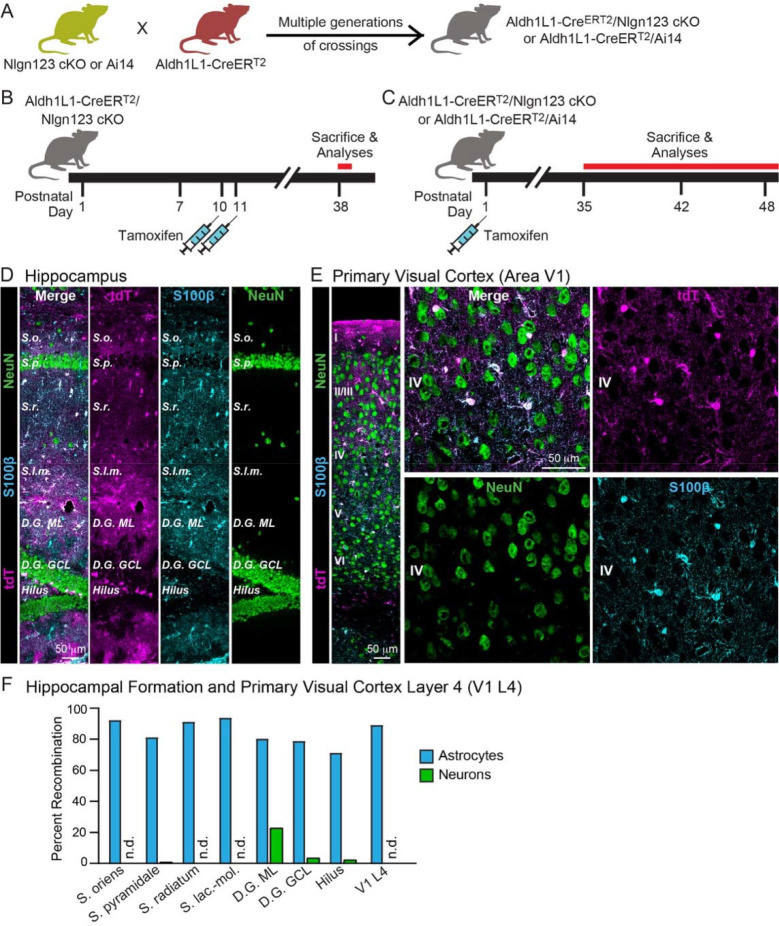
*Nlgn1*-*3* are efficiently and selectively deleted in astrocytes by crossing triple *Nlgn1–3* conditional KO mice with *Adh1l1*-CreER^T2^ driver mice and inducing Cre-activity with tamoxifen early during postnatal development (**A**) Breeding strategy. Triple conditional KO mice carrying floxed *Nlgn1*, *Nlgn2*, and *Nlgn3* alleles or mice with a Cre-sensitive tdTomato (tdT) reporter allele (Ai14) were crossed with pan-astrocyte, tamoxifen-inducible Aldh1l1-CreER^T2^ BAC transgenic mice. *Nlgn1–3* cKO mice were crossed for multiple generations until homozygosity was reached (females: Nlgn1^f/f^ 2^f/f^ 3^f/f^, males: Nlgn1^f/f^ 2^f/f^ 3^f/y^). (**B** & **C**) Two different tamoxifen-induced Cre-activation schedules were used to delete *Nlgn1–3* in astrocytes. Aldh1l1-CreER^T2^ mice and controls (littermate Nlgn1–3 cKO mice lacking the Aldh1l1-CreER^T2^ allele) were injected with tamoxifen at P10 and P11 (**B**) (Trotter et al., 2021) or at P1 (**C**). Mice were sacrificed at least four (B) or five weeks post Cre induction to ensure complete deletion of neuroligins and decay of any astrocyte-specific neuroligin proteins. (**D** & **E**) To confirm the specificity and efficiency of the deletion of target genes in astrocytes using the Aldh1l1-CreER^T2^ BAC transgenic mouse line via tamoxifen injection at P1, Cre-recombination was visualized in the hippocampus (**D**) and primary visual cortex (**E**) via expression of tdT in reporter mice (magenta). Sections were additionally labeled for NeuN to mark neurons (green) and S100β to mark astrocytes (blue). (**F**) The Algh1l1-CreER^T2^-induced deletion of floxed genes produced by P1 tamoxifen injections is effective and selective for astrocytes as quantified using expression of tdTomato in reporter mice. tdTomato expression was quantified in the CA1 region of the hippocampus (the *S. oriens, S. pyramidale, S. radiatum,* and *S. lacunosum-moleculare*), the dentate gyrus (molecular layer [ML], granule cell layer [GCL], and hilus), and layer IV of the primary visual cortex.

To assess the fundamental functions of astrocytic neuroligins in synaptogenesis and astrocyte development, we generated mice that enable the inducible conditional deletion of astrocytic *Nlgn1*, *Nlgn2*, and *Nlgn3*. We targeted the conditional deletion specifically to astrocytes by crossing Aldh1l1-CreER^T2^ BAC transgenic mice that exhibit astrocyte-specific expression of tamoxifen-activatable Cre-recombinase (Winchenbach et al., 2016; Yu et al., 2020) with *Nlgn1*-*3* conditional KO (cKO) mice (Zhang et al., 2015) (Figure 2A). We then bred homozygous female triple *Nlgn1–3* cKO mice with male triple cKO mice carrying the Aldh1l1-CreER^T2^ allele to generate littermate male and female Cre-positive test mice and Cre-negative control mice. Mice were injected with tamoxifen intraperitoneally at postnatal days 10 and 11 (P10 and P11) or subcutaneously at postnatal day 1 (P1), and were analyzed at P35 – P48 (Figure 2B, 2C). These two Cre activation timepoints were chosen because manipulation of astrocytic neuroligins at P10–11 has been previously reported to reduce astrocyte morphogenesis and excitatory synaptogenesis (Stogsdill et al. 2017), and because we also wanted to ensure that we could capture a possible earlier function of astrocytic neuroligins during the major phase of developmental synaptogenesis that occurs around P6–12 in mice.

To confirm the efficacy of CreER^T2^ induction, we focused on the subcutaneous tamoxifen injections at P1 since this condition was used for most of our experiments as the most rigorous approach and was not validated in detail previously. We crossed Aldh1l1-CreER^T2^ BAC transgenic mice with Cre-dependent tdTomato (tdT) reporter mice (Ai14), injected the resulting double-transgenic mice subcutaneously with tamoxifen at P1, and analyzed the mice at P35 by staining brain sections for tdTomato (Figure 2C). In the CA1 region of the hippocampus of these mice, we observed tdTomato-labeling in over 80% of astrocytes in the *S. oriens*, *S. pyramidale*, *S. radiatum*, or *S. lacunosum*-*moleculare*. We found no neuronal recombination except for low levels in the dentate gyrus that is populated by adult-born neurons (Figure 2D & 2F). In layer IV (L4) of the primary visual cortex (V1), we also detected Cre-dependent tdTomato expression only in astrocytes without non-specific recombination in neurons. Again, approximately 90% of astrocytes expressed tdTomato as an indicator of Cre activity (Figure 2E & 2F). Thus, the P1 tamoxifen injection protocol efficiently activates Cre recombinase in Aldh1l1-CreER^T2^ BAC transgenic mice.

### Deletion of astrocytic neuroligins has no effect on synaptic proteins

**Figure 3:**
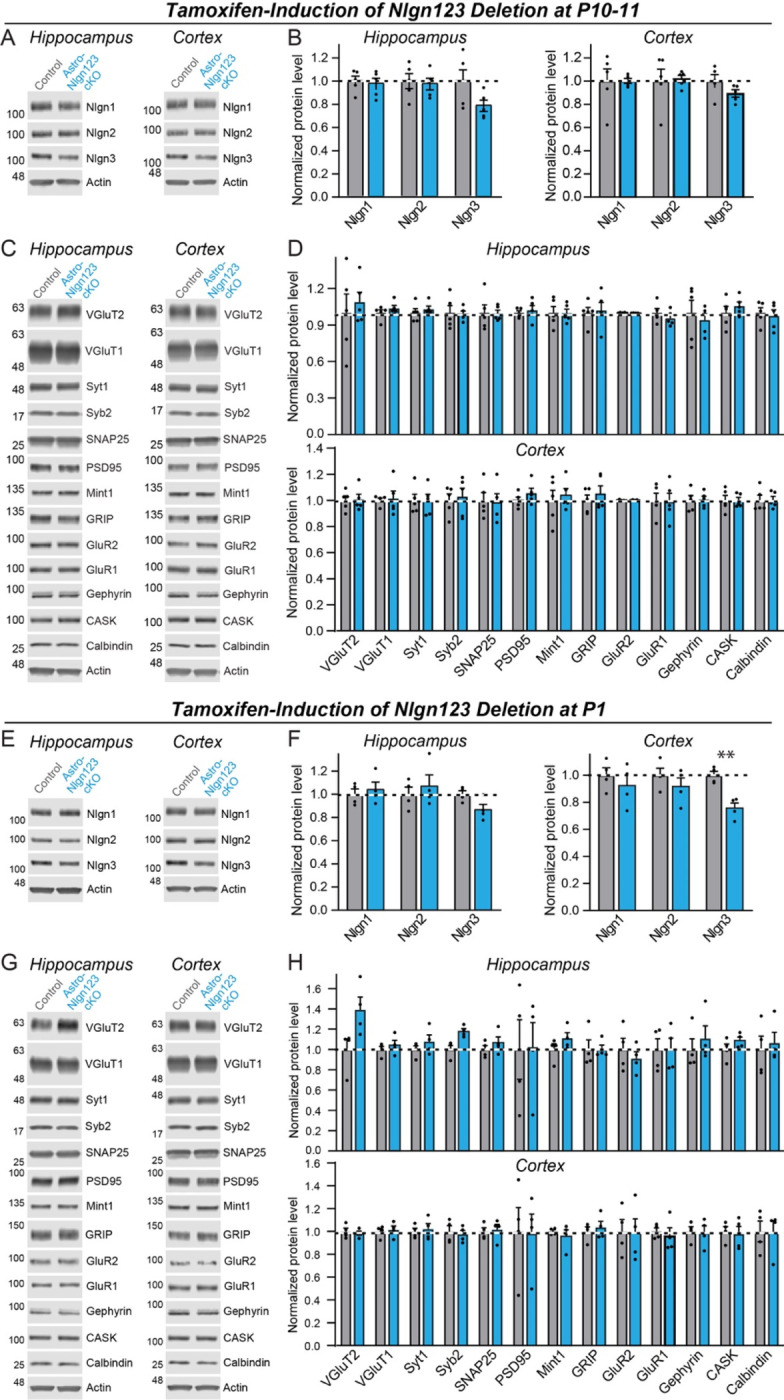
Conditional deletion of *Nlgn1*-*3* in astrocytes throughout the brain at early postnatal timepoints (P10/11 or P1) has only modest effects on overall neuroligin protein levels and does not significantly alter the synaptic proteome (**A** & **B**) Representative immunoblots and quantifications of *Nlgn1*, *Nlgn2*, and *Nlgn3* protein levels from hippocampal (**A**) and cortical lysates (**B**) of astrocyte *Nlgn1–3* cKO and littermate control mice injected with tamoxifen at P10 and P11 and sacrificed at P38. Proteins were quantified on immunoblots using fluorescent secondary antibodies, with protein levels normalized to β-actin and then to control levels (n = 5, all male). (**C** & **D**) Representative immunoblots and (**D**) quantification for various synaptic protein levels from hippocampal and cortical lysates of astrocyte *Nlgn1–3* cKO and littermate control mice injected with tamoxifen at P10 and P11 and sacrificed at P38. Protein is quantified using fluorescent secondary antibodies, with protein levels normalized to β-actin and then to control levels (n = 5, all male). (**E-H**) Same as (**A-D**) except mice were injected with tamoxifen at P1 and sacrificed at P35 (n = 4, 2 male & 2 female). Numerical data are means ± SEM with statistical significance determined by unpaired two-tailed t-test (**, p<0.01).

As a first step towards testing whether astrocytic neuroligins are essential for synaptogenesis in the hippocampus and cortex, we screened for changes in synaptic protein levels in mice with astrocyte-specific deletions of *Nlgn1–3*. We collected hippocampal and cortical lysates from the brains of *Nlgn1–3* cKO mice with activation of Cre-recombinase in astrocytes (referred to as “astro-*Nlgn123* cKO mice”) and from their Cre-negative littermates after induction of Cre-recombinase at P10 and P11 (Figure 3A–D) or at P1 (Figure 3E–H). Lysates were collected at P35–38 and analyzed by quantitative immunoblotting using fluorescent secondary antibodies. Both the levels of synaptic proteins and of neuroligins were measured in comparison to loading controls.

We found that the levels of *Nlgn1*, *Nlgn2*, and *Nlgn3* proteins were not detectably decreased in either the cortex or the hippocampus after either P10–11 or P1 induction of Cre-recombinase, except for a significant decline (~25%) in *Nlgn3* protein levels in cortical lysates after the P1 induction (Figure 3A, 3B, 3E and 3F). Given that astrocytes account for only ~20% of cells in brain, the absence of a significant decrease in neuroligin proteins in brain after the *Nlgn1–3* deletion in astrocytes is not surprising as one would expect maximally a ~20% decrease in neuroligin levels if mRNA and protein levels perfectly correlated. Protein level measurements are inherently noisy, making detections of change of <20% difficult. The finding of a ~25% decrease in *Nlgn3* levels in cortex after the P1 induction (Figure 3F) is consistent with this assessment since *Nlgn3* is the most abundantly expressed neuroligin isoform in astrocytes (Figure 1).

We next quantified the levels of selected synaptic proteins as a function of the astrocytic *Nlgn1–3* deletion (Figure 3C, D, G, and H). We analyzed 12 synaptic proteins as well as calbindin as a marker of subsets of inhibitory neurons. No significant changes in any protein analyzed were detected in hippocampus or cortex.

### Astrocytic neuroligins are dispensable for hippocampal synapse formation

**Figure 4:**
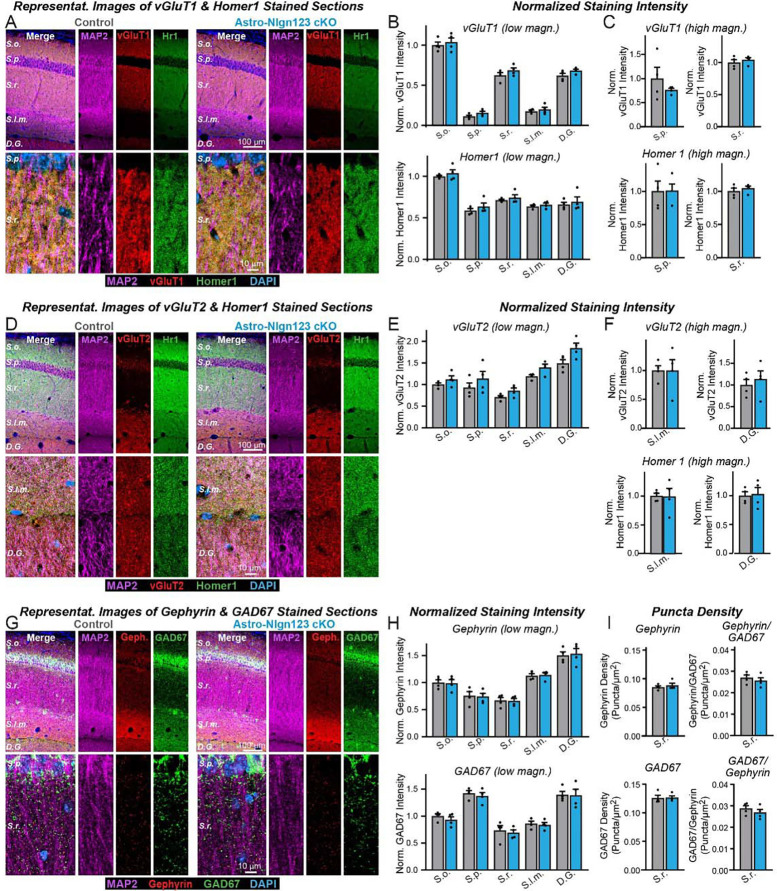
Conditional deletion of *Nlgn1*-*3* in astrocytes starting at P1 does not alter excitatory or inhibitory synapse numbers in the hippocampus as assessed by immunocytochemistry with antibodies to synaptic markers (**A**) Representative images of CA1 and dentate gyrus hippocampal sections from astrocyte Nlgn1–3 cKO and littermate control mice, injected with tamoxifen at P1 and sacrificed at P35, stained for dendritic marker MAP2 (magenta), excitatory presynaptic marker vGluT1 (red), excitatory postsynaptic marker Homer1 (green), and DAPI (blue), taken at 20X (top) and 60X (bottom) magnification. (**B**) Quantification of total vGluT1 (top) and Homer1 (bottom) immunofluorescence signal for low magnification imaging (20X) across the layers of the hippocampus (*S. oriens, S. pyramidale, S. radiatum, S. lacunosum-moleculare,* dentate gyrus molecular layer), first internally normalized to MAP2 and then to average vGluT1 (top) or Homer1 (bottom) immunofluorescence level in *S. oriens* of control mice. (**C**) Quantification of total vGluT1 (top) and Homer1 (bottom) immunofluorescence signal for high magnification imaging (60X) in the CA1 *S. pyramidale* (left) and *S. radiatum* (right), first internally normalized to MAP2 and then to average vGluT1 (top) or Homer1 (bottom) immunofluorescence level in control mice. (**D**) Representative images of CA1 and dentate gyrus hippocampal sections from astrocyte Nlgn1–3 cKO and littermate control mice stained for dendritic marker MAP2 (magenta), excitatory presynaptic marker vGluT2 (red), excitatory postsynaptic marker Homer1 (green), and DAPI (blue), taken at 20X (top) and 60X (bottom) magnification. (**E**) Quantification of total vGluT2 immunofluorescence signal for low magnification imaging (20X) across the layers of the hippocampus, first internally normalized to MAP2 and then to average vGluT2 immunofluorescence level in *S. oriens* of control mice. (**F**) Quantification of total vGluT2 (top) and Homer1 (bottom) immunofluorescence signal for high magnification imaging (60X) in the CA1 *S. lacunosum-moleculare* (left) and dentate gyrus molecular layer (right), first internally normalized to MAP2 and then to average vGluT2 (top) or Homer1 (bottom) immunofluorescence level in control mice. (**G**) Representative images of CA1 and dentate gyrus hippocampal sections from astrocyte *Nlgn1–3* cKO and littermate control mice stained for dendritic marker MAP2 (magenta), inhibitory postsynaptic marker Gephyrin (red), inhibitory presynaptic marker GAD67 (green), and DAPI (blue), taken at 20X (top) and 60X (bottom) magnification. (**H**) Quantification of total Gephyrin (top) and GAD67 (bottom) immunofluorescence signal for low magnification imaging (20X) across the layers of the hippocampus, first internally normalized to MAP2 and then to average Gephyrin (top) or GAD67 (bottom) immunofluorescence level in *S. oriens* of control mice. (**I**) Quantification of puncta density for Gephyrin (top left), GAD67 (bottom left), Gephyrin having GAD67 (top right), and GAD67 having Gephyrin (bottom right) for high magnification imaging (60X) in the CA1 *S. radiatum*. Data are means ± SEM with statistical significance determined by unpaired two-tailed t-test (n=4, 2 male & 2 female).

Measurements of synaptic proteins are a relatively insensitive approach to assessing synapse numbers. For a more direct measurement, we labeled cryosections from the brains of littermate astro-*Nlgn123* cKO and control mice at P35 after P1 induction of Cre-recombinase with antibodies to the excitatory presynaptic marker vGluT1, the excitatory postsynaptic marker Homer1 and the dendritic marker MAP2, and counterstained the sections with DAPI (Figure 4A). Because excitatory synapses in the hippocampus are too dense to be individually resolved by confocal imaging, we used the overall staining intensity as a proxy for synapse density. Low magnification (20x) imaging across the layers of the CA1 region of the hippocampus and the molecular layer of the dentate gyrus revealed no effect of the astro-*Nlgn123* cKO on vGluT1 or Homer1 staining intensity (Figure 4B). In order to increase the signal-to-noise ratio and improve our ability to detect small changes in staining intensity, we additionally performed high-magnification (60x) confocal imaging of the *S. pyramidale* and *S. radiatum* in the CA1 region (Figure 4A). Again, vGluT1 and Homer1 staining intensities were not altered by loss of astrocytic *Nlgn1*-3 at P1 (Figure 4A, C). Next, we stained hippocampal sections with antibodies to the excitatory presynaptic marker vGlut2 alongside Homer1 and MAP2 since vGluT2 is present only in a subset of excitatory synapses (Figure 4D). We also detected no changes in the vGluT2 staining intensity at either low or high magnifications (Figure 4E, F).

To assess whether astrocytic neuroligins are required for inhibitory synapse formation, we labeled hippocampal sections with antibodies to the inhibitory presynaptic marker GAD67, the inhibitory postsynaptic marker gephyrin and MAP2 and counterstained the sections with DAPI (Figure 4G). Again, no changes in gephyrin or GAD67 staining intensity were observed after P1 deletion of astrocytic *Nlgn1–3* using low or high magnification imaging (Figure 4H, S2A). Given the lower density of inhibitory synapses in the hippocampus, individual inhibitory synapses could be resolved with high magnification imaging. This enabled us to quantify the density and size of the inhibitory GAD67 and Gephyrin synaptic puncta in the CA1 *S. radiatum*. Both were not changed in astro-*Nlgn123* cKO mice compared to control mice (Figure 4I, left panels and S2B). Finally, the number of synapses containing matched pre- (GAD67) and postsynaptic signals (gephyrin) was also quantified but exhibited no change in astro-*Nlgn123* cKO mice (Figure 4I, right panels). Thus, astrocytic *Nlgn1–3* are not essential for either excitatory or inhibitory synaptogenesis in the hippocampus.

### Astrocytic neuroligins are not required for basal synaptic function

**Figure 5:**
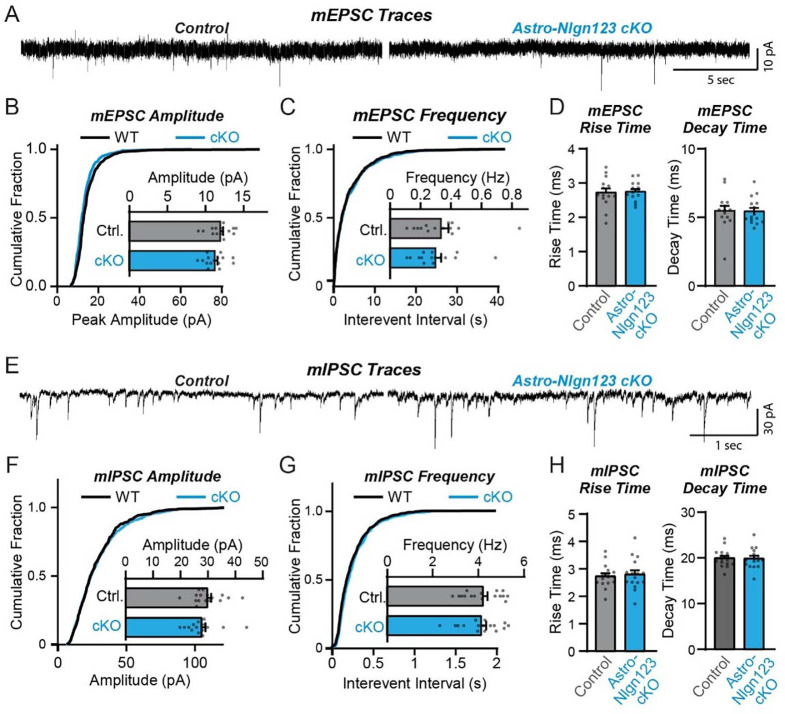
Conditional deletion of *Nlgn1*-*3* in astrocytes starting at P1 has no major effect on basal excitatory or inhibitory neurotransmission monitored in hippocampal CA1 pyramidal neurons (**A**) Representative traces for miniature EPSCs (mEPSCs) from CA1 pyramidal neurons in acute slices from astrocyte *Nlgn1–3* cKO and littermate controls injected with tamoxifen at P1 and recorded at P44 - P50. (**B**) Cumulative distribution and summary graph of mEPSC amplitude and (**C**) frequency. (**D**) Summary graph of mEPSC rise (left) and decay (right) times (n = 14–15 cells / 3 mice per genotype). (**E-H**) Same as (**A-D**) except for miniature IPSCs (mIPSCs) (n = 16 cells / 3 mice per genotype). Data in summary graphs are means ± SEM with each data point representing individual cells. Unpaired two-tailed t-tests were used to test statistical significance of data in bar graphs, and Kolmogorov-Smirnov tests were used for cumulative curves (****, p<0.0001).

The lack of a requirement of astrocytic neuroligins for synapse formation in the hippocampus agrees well with previous data demonstrating that constitutive neuroligin deletions do not decrease synapse numbers, but severely impair synaptic transmission (Varoqueaux et al., 2006; Chubykin et al., 2007). To test whether loss of astrocytic *Nlgn1–3* causes a functional impairments of synapses, we monitored spontaneous excitatory and inhibitory synaptic transmission in CA1 region pyramidal neurons. We produced acute slices from littermate astro-*Nlgn123* cKO and control mice at P44–50 after P1 tamoxifen injections and performed patch-clamp recordings from CA1-region pyramidal neurons in the presence of tetrodotoxin (Figure 5). The amplitude, frequency, and kinetics of mEPSCs and mIPSCs were not changed by deletion of astrocytic *Nlgn1–3* (Figure 5). Furthermore, deletion of astrocytic neuroligins did not alter the membrane properties of CA1 pyramidal neurons (Figure S3). These data suggest that astrocytic neuroligins are not essential for basal synaptic transmission of CA1 pyramidal neurons, whereas previous data demonstrated that neuronal neuroligins are (Földy et al., 2015; Chanda et al., 2018).

### Astrocytic neuroligins are not essential for synapse formation in the visual cortex

**Figure 6:**
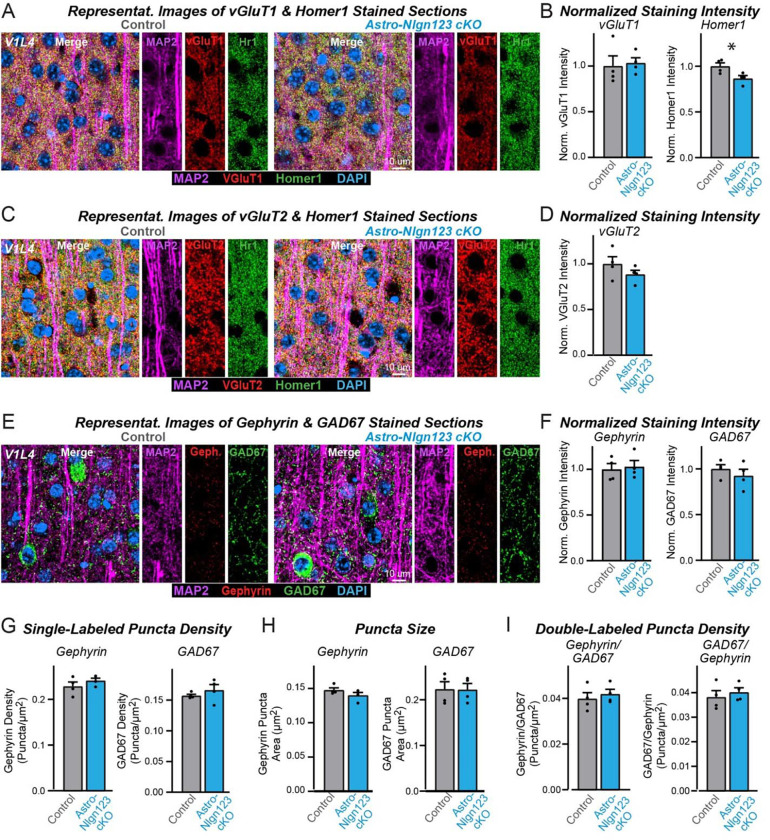
Conditional deletion of *Nlgn1*-*3* in astrocytes starting at P1 does not alter excitatory or inhibitory synapse numbers in layer IV of the primary visual cortex as assessed by immunocytochemistry with antibodies to synaptic markers (**A**) Representative images of primary visual cortex (V1) layer IV (L4) astrocyte *Nlgn1–3* cKO and littermate control mice, injected with tamoxifen at P1 and sacrificed at P35, stained for dendritic marker MAP2 (magenta), excitatory presynaptic marker vGluT1 (red), excitatory postsynaptic marker Homer1 (green), and DAPI (blue) taken at 60X magnification. (**B**) Quantification of total vGluT1 (left) and Homer1 (right) immunofluorescence signal in V1L4 first internally normalized to MAP2 and then to average vGluT1 (left) or Homer1 (right) immunofluorescence level in control mice. (**C**) Representative images of V1L4 astrocyte *Nlgn1–3* cKO and littermate control mice stained for dendritic marker MAP2 (magenta), excitatory presynaptic marker vGluT2 (red), excitatory postsynaptic marker Homer1 (green), and DAPI (blue) taken at 60x magnification. (**D**) Quantification of total vGluT2 immunofluorescence signal in V1L4 first internally normalized to MAP2 and then to average vGluT2 immunofluorescence level in control mice. (**E**) Representative images of V1L4 astrocyte *Nlgn1–3* cKO and littermate control mice stained for dendritic marker MAP2 (magenta), inhibitory postsynaptic marker Gephyrin (red), inhibitory presynaptic marker GAD67 (green), and DAPI (blue) taken at 60X magnification. (**F**) Quantification of total Gephyrin (left) or GAD67 (right) immunofluorescence signal in V1L4 first internally normalized to MAP2 and then to average gephyrin (left) or GAD67 (right) immunofluorescence level in control mice. (**G**) Quantification of Gephyrin (left) or GAD67 (right) puncta density. (**H**) Quantification of Gephyrin (left) or GAD67 (right) puncta size in V1L4. (**I**) Quantification of puncta density for Gephyrin having GAD67 (left) or GAD67 having Gephyrin (right) in V1L4. Data are means ± SEM with statistical significance determined by unpaired two-tailed t-test (*, p<0.05) (n=4, 2 male & 2 female).

Since the finding of Stogsdill et al. (2017) of an essential function for astrocytic neuroligins in excitatory synapse formation was obtained in the visual cortex and it is possible that the function of astrocytic neuroligins differs between the hippocampus and the visual cortex, we explored the consequences of the genetic deletion of astrocytic *Nlgn1–3* on synapses in the visual cortex. We obtained cryosections of the visual cortex containing area V1 from 11stro-*Nlgn123* cKO and littermate control mice at P35 after the mice had been injected with tamoxifen at P1. The sections were co-stained with antibodies to Homer1, MAP2, and either vGluT1 or vGluT2 and counterstained with DAPI. We then imaged layer 4 (L4) of area V1 of the visual cortex, the same layer used by Stogsdill et al. (2017) (Figure 6A and 6C). As in the hippocampus, excitatory synapses in L4 of the primary visual cortex are too dense to be resolved individually, so we quantified the overall staining intensity as a proxy for synapse number. High magnification imaging revealed that loss of astrocytic neuroligins had no effect on the staining intensity of vGluT1 or vGluT2, but revealed a small decrease (~15%) in the staining intensity for Homer1 (Figure 6B & 6D).

To assess the effect of deleting astrocytic neuroligins on inhibitory synaptogenesis in L4, sections were co-stained with antibodies to GAD67, gephyrin, and MAP2 and again counterstained with DAPI (Figure 6E). The staining intensity of GAD67 and gephyrin were unaffected by the astrocytic neuroligin deletion (Figure 6F). Since inhibitory puncta can be resolved with confocal imaging, we also quantified the density and size of GAD67 and gephyrin puncta (Figure 6G, H), as well as the density of colocalized GAD67-gephyrin puncta (Figure 6I). Deletion of astrocytic *Nlgn1–3* caused no change in any of these measures. These data suggest that, in our hands, astrocytic neuroligins are not fundamentally required for synaptogenesis in layer 4 of the primary visual cortex.

### Astrocytic neuroligins are not essential for astrocyte morphogenesis

**Figure 7:**
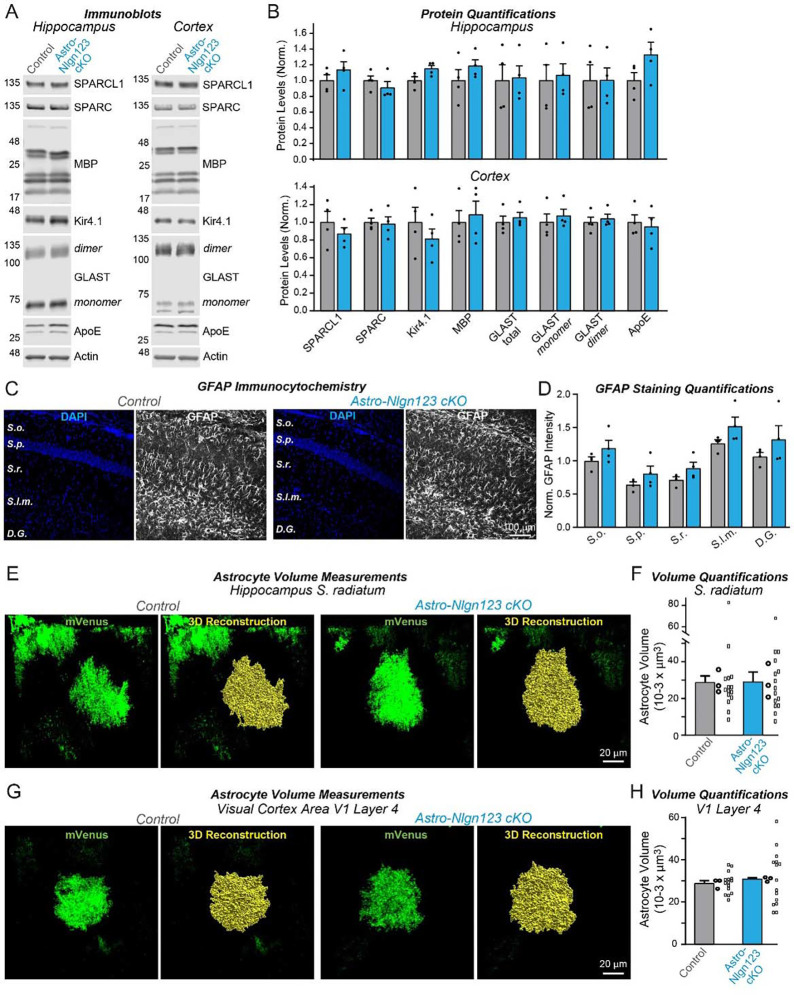
Conditional deletion of *Nlgn1*-*3* in astrocytes starting at P1 does not detectably alter the proteome or the cytoarchitecture of astrocytes in the hippocampus or layer IV of the primary visual cortex (**A**) Representative immunoblots and (**B**) quantification for various glial protein levels from hippocampal and cortical lysates of astrocyte *Nlgn1–3* cKO and littermate control mice injected with tamoxifen at P1 and sacrificed at P35. Protein is quantified using fluorescent secondary antibodies, with protein levels normalized to β-actin and then to control levels (n = 4, 2 male & 2 female). (**C**) Representative images of CA1 and dentate gyrus hippocampal sections from astrocyte *Nlgn1–3* cKO and littermate control mice, injected with tamoxifen at P1 and sacrificed at P35, stained for astrocytic marker GFAP (white) and DAPI (blue), taken at 20X magnification. (**D**) Quantification of total GFAP immunofluorescence signal across the layers of the hippocampus (*S. oriens, S. pyramidale, S. radiatum, S. lacunosum-moleculare,* dentate gyrus molecular layer), normalized to average GFAP immunofluorescence level in *S. oriens* of control mice (n = 4, 2 male & 2 female). (**E**) To measure astrocyte volume, astrocyte *Nlgn1–3* cKO and littermate control mice were injected with tamoxifen at P1, underwent stereotactic injections of AAV expressing membrane-bound mVenus in astrocytes in the hippocampus at P14, and were subsequently sacrificed two weeks later at P35. Representative images of mVenus-expressing astrocytes in CA1 *S. radiatum* are shown with corresponding 3D volume reconstruction performed in Imaris. (**F**) Summary graph of CA1 *S. radiatum* astrocyte volumes shown averaged per animal with means ± SEM on the bar graph, as well as data points for individual astrocyte volumes. Statistical significance determined by unpaired two-tailed t-test of data averaged per animal (n=3, 1 male & 2 female). (**G & H**) Same as E & F, except for primary visual cortex layer IV astrocytes. Numerical data are means ± SEM. Dots in bar graphs represent independent biological replicates; in F and H, larger dots are independent biological replicates and smaller dots are pseudoreplicates since these are commonly reported in papers to boost statistical significance.

It is possible that astrocytic neuroligins could be involved in astrocyte morphogenesis even if they are not contributing to synapse formation. Thus, we asked whether astrocytic neuroligins contribute to the morphogenesis of astrocytes and their complex cytoarchitecture. We first measured the levels of a series of glial proteins in lysates of the hippocampus and cortex of astro-*Nlgn123* cKO and control mice that had been injected with tamoxifen at P1 and were analyzed at P35 (Figure 7A). However, we failed to uncover major changes (Figure 7B). Next, we immunostained astrocytes in CA1 hippocampal sections for glial fibrillary acidic protein (GFAP) that is constitutively expressed in mouse hippocampal astrocytes but did not detect any alterations in GFAP expression in astro-*Nlgn123* cKO mice at P35 (Figure 7C, D).

Finally, to directly test the claim that astrocytic neuroligins control astrocyte size (Stogsdill et al. 2017), we applied tamoxifen to astro-*Nlgn123* cKO and control mice at P1 and stereotactically injected AAVs expressing membrane-targeted mVenus under control of the GFAP promoter into their hippocampus or primary visual cortex at P21. We then imaged relatively thick sections (100 μm) from the hippocampus or primary visual cortex of these mice by confocal microscopy and reconstructed the entire volumes of astrocytes in the *S. radiatum* of the hippocampal CA1 region and in L4 of the primary visual cortex (Figure 7E, G). Quantifications of these volumes did not uncover any differences between astro-*Nlgn123* cKO and control mice, indicating that deletion of astrocytic *Nlgn1–3* did not alter astrocyte size (Figure 7F, H).

## DISCUSSION

Astrocytes are integral to shaping neural circuits. In the gray matter of the cerebral cortex (Halassa et al. 2007), hippocampus (CA1 *S. radiatum*) (Bushong et al. 2001), and cerebellar cortex (Spacek 1985), astrocytes (or their cousins, the Bergmann glia in the cerebellum) occupy non-overlapping territories, effectively “tiling” the neuropil. Within their territories, astrocytes not only contact all other cells in brain, but also associate with axons and dendrites and ensheath synaptic junctions, thus forming tripartite synapses (Verkhratsky and Nedergaard, 2018; Saint-Martin and Goda, 2022; Oliveira and Araque, 2022; Lyon and Allen, 2022; Arizono and Nägerl, 2022). Astrocytes exhibit an exceptionally complex cytoarchitecture that includes thousands of fine processes infiltrating the neuropil where these processes likely perform multiple essential functions. At tripartite synapses, astrocyte functions include the removal of released glutamate from the perisynaptic area via glutamate transporters, thereby facilitating input specificity and preventing neurotoxicity (Ventura and Harris 1999). Astrocytes express numerous neurotransmitter receptors, enabling them to contribute to synaptic signaling and to respond to synaptic activity in a dynamic and activity-dependent manner (Allen and Eroglu, 2017). Moreover, astrocytic processes contain an array of ion channels that regulate their activity and contribute to ionic homeostasis not only at synapses but also at other cellular locations, such as the nodes of Ranvier (Verkhratsky and Nedergaard, 2018; Black and Waxman, 1988) and blood vessels (Attwell et al., 2010). The molecular mechanisms by which astrocytes interact with synapses and other brain components, however, are incompletely understood.

Recent transcriptomic studies revealed that, in addition to neurotransmitter receptors and ion channels, astrocytes express multiple cell-adhesion molecules that are known to function at synapses, suggesting that synaptic cell-adhesion molecules may mediate the integration of astrocytes into tripartite synapses and that astrocytes may regulate synapses via signals that are transmitted by such cell-adhesion molecules (Saint-Martin and Goda, 2022; Tan and Eroglu, 2021). Indeed, our analysis of multiple independent RNAseq datasets showed that astrocytes express high levels in particular of neuroligins, canonical postsynaptic cell-adhesion molecules that are known to be essential for synaptic function (Figure 1). The absolute and relative expression levels of different neuroligin isoforms in astrocytes varied among studies, but all studies concurred in the conclusion that the mRNA levels of the three major neuroligins (*Nlgn1–3*) are overall similar in astrocytes and neurons (Figure 1; *Nlgn4* was not captured in the RNAseq studies owing to its low abundance and/or the high GC content of its mRNA). Thus, neuroligins are clearly not neuron-specific, which is consistent with the possibility that neuroligins in astrocytes function to embed astrocytes in tripartite synapses and to enable astrocytes to contribute to the formation and performance of synapses, a hypothesis that was proposed in a pioneering paper by Stogsdill et al. (2017).

Motivated by this paper that has since guided all reviews on the subject, we here investigated the function of neuroligins expressed in astrocytes. We aimed to achieve two related goals. First, to test the hypothesis inherent in the Stogsdill et al. (2017) paper that neuroligins as synaptic cell-adhesion molecules perform a critical role in astrocytes by promoting synapse formation. Second, to address a major contradiction arising from a comparison of the Stogsdill et al. (2017) paper with previous studies. Specifically, Stogsdill et al. (2017) showed that astrocytic neuroligins in general are essential for excitatory synapse formation and for the normal morphology of astrocytes, and that *Nlgn2* in particular is required for excitatory synapse formation. Inconsistent with this conclusion, however, numerous earlier studies detected *Nlgn2* protein only in inhibitory but not in excitatory synapses (Graf et al., 2004; Varoqueaux et al., 2004; Panzanelli et al., 2017; Hoon et al., 2009; Patrizi et al., 2008; Takacs et al., 2013 and 2018). Moreover, extensive earlier experiments showed that constitutive deletion of *Nlgn2* in both neurons and glia caused a dysfunction of only inhibitory but not of excitatory synapses (Vareauqueaux et al., 2006; Chubykin et al., 2007).

To investigate whether neuroligins expressed in astrocytes, as opposed to neuroligins expressed in neurons, perform an essential function in synapse formation, we used a rigorous genetic approach. We generated a tamoxifen-inducible mouse model that enables conditional deletion of *Nlgn1–3* in all astrocytes (Figure 2A) and analyzed the effect of such deletion in depth in the hippocampal CA1 region and the visual cortex. Using two different time points of induction of the deletion of astrocytic neuroligins (P1 and P10–11; Figure 2), we made two principal observations.

First, deletion of *Nlgn1–3* from astrocytes has no detectable effect on the density of excitatory or inhibitory synapses in the hippocampus or cortex, or on the properties of spontaneous excitatory or inhibitory synaptic transmission in the hippocampus. We established this conclusion by extensive immunocytochemical analyses using a panel of antibodies to synaptic markers (Figure 4, 6), by electrophysiology (Figure 5), and by quantification of the synaptic proteome (Figure 3). Thus, astrocytic *Nlgn1–3* are not required for synapse formation or maintenance.

Second, deletion of *Nlgn1–3* from astrocytes does not cause a measurable change in the cytoarchitecture of astrocytes (Figure 7). This was assessed by monitoring the astrocyte protein composition, GFAP staining in the hippocampus, and three-dimensional reconstruction of astrocytes in the hippocampus or cortex. Thus, *Nlgn1–3* are not required for the morphogenesis or cell shape maintenance of astrocytes.

Two questions arise at this point. First, is it possible that the genetic deletion of *Nlgn1–3* in astrocytes for some reason was ineffective? To assess the efficiency of the tamoxifen-induced deletion of floxed genes, we quantified Cre-activity using the P1 induction protocol, which is the approach used for most experiments in this paper since it aims to permanently delete (not just knock down) neuroligins at a time early in development (Figure 2D–F). We observed Cre-mediated recombination in >80% of astrocytes but in <5% of neurons except for the dentate gyrus in which neurons are continuously replenished by adult neurogenesis. This finding confirms that the genetic approach efficiently induces Cre-recombinase activity, which in turn we previously demonstrated using the same conditional triple *Nlgn1–3* alleles employed here quantitatively deletes all three neuroligin genes (Chanda et al., 2017; Zhang et al., 2015).

Second, how can we explain that our results are inconsistent with those of the pioneering study of Stogsdill et al. (2017), although they are consistent with other previous studies? There are multiple differences between our approach and that of Stogsdill et al. (2017) that may contribute to the differences in results. We used a purely genetic approach, whereas most data in the Stogsdill et al. (2017) paper were obtained with RNA-interference (RNAi). RNAi is prone to side effects because of off-target effects and, more importantly, because RNAi interferes with the entire microRNA processing machinery of a cell.

However, Stogsdill et al. (2017) also used conditional *Nlgn2* KO mice (the same alleles that we originally generated and that were used in the present study) for analyses of spontaneous synaptic transmission. Specifically, Stogsdill et al. (2017) employed the tamoxifen-induced deletion of astrocytic *Nlgn2* at P10–11 using the GLAST-CreER^T2^ mouse line, which led led to an impairment in the function of excitatory synapses in the layer V of the primary visual cortex (Stogsdill et al. 2017). This suggests that at least for some of their findings, off-target effects by RNAi cannot completely explain the differences with our results. Another feature in which the approach of Stogsdill et al. (2017) differs from ours is that Stogsdill et al. (2017) largely employed ‘sparse’ deletions of neuroligins in a subset of astrocytes, whereas we used a global deletion of neuroligins in nearly all astrocytes. The phenotypes of sparse deletions is often difficult to interpret functionally because a mutant cell with a deletion can be at a competitive disadvantage with surrounding wild-type cells, leading to changes that are not directly related to the function of the deleted gene but are caused by this competitive disadvantage. It is thus possible, maybe even likely, that neuroligins perform a non-synaptic function in astrocytes, and that owing to indirect downstream effects induced by the competition with surrounding wild-type astrocytes, the shape of the mutant astrocytes and the formation of excitatory synapses may have been compromised even though astrocytic neuroligins are not important for the formation of synapses.

Astrocytes tile the neuropil by engaging in competition with neighboring astrocytes for individual territories through a mechanism similar to that of dendritic tiling (Freeman 2010). Thus, we might expect that a sparse manipulation would produce an exaggerated phenotype over that of a global manipulation. An indirect effect produced by a competitive disadvantage induced with a sparse deletion has been recently reported in astrocytes for hepatocyte cell-adhesion molecule (hepaCAM). In this study, sparse deletion of hepaCAM led to a reduction in the volume of the astrocyte territory, while global deletion of hepaCAM had no effect (Baldwin et al. 2021). Additionally, it has been reported that while global deletion of Nlgn1 *in vivo* has no effect on synapse number in cortical layer 2/3 pyramidal neurons, sparse deletion via electroporation of shRNA targeting *Nlgn1* results in a reduction of spine density and synapse number, although it was unclear from that study whether the shRNA acted by a *Nlgn1*-specific mechanism (Kwon et al., 2012).

A further difference between our study and that of Stogsdill et al. (2017) is the timepoint of quantification. Our analyses were performed at P35–42, whereas Stogsdill et al. (2017) examined synapses at P21, prior to completion of synaptic and astrocytic morphological refinement (Freeman 2010). Thus, it is possible that the phenotype observed by Stogsdill et al. (2017) is developmentally transient in nature and recovers upon completion of brain maturation. A transient effect of this type has been described for loss of astrocytic *Nlgn2* in Drosophila. Here, RNAi knockdown of astrocytic *Nlgn2* delayed motor circuit closure during development but did not result in robust, lasting behavioral phenotypes after the critical period had passed (Ackerman et al. 2021). It is important to note that, in agreement with our data indicating that astrocytic neuroligins do not control astrocyte morphogenesis, astrocyte-specific deletion of *Nlgn2* in drosophila has also been shown to have no effect on astrocyte volume or tiling (Ackerman et al. 2021). Furthermore, in a screen investigating astrocyte diversity across the nervous system, neuroligins were not identified among the genes driving differences in astrocyte morphology (Endo et al. 2022).

In summary, it seems likely that expression of *Nlgn1–3* in astrocytes has no direct function in synapse formation or in shaping the cytoarchitecture of astrocytes, but that *Nlgn1–3* perform other important roles in astrocytes that remain to be identified. Continual advances in the tools available to access astrocytes, in concert with genetic models that allow temporally-defined manipulation of genes, will be key to discovering these roles that may provide new insights into astrocyte biology.

## EXPERIMENTAL PROCEDURES

### Mice.

Aldh1l1-Cre/ER^T2^ BAC transgenic mice were purchased from The Jackson Laboratory (Strain #:029655, RRID:IMSR_JAX:029655) and were bred with *Nlgn1*-*3* cKO mice, generated as previously described (Zhang et al., 2015), until homozygosity was reached for the floxed *Nlgn1–3* alleles. Litters resulting from crossing female *Nlgn1–3* cKO mice with male triple cKO mice carrying the Aldh1l1-CreER^T2^ were injected with tamoxifen at early postnatal timepoints to generate astro-*Nlgn123* cKO mice and littermate controls for experiments. After weaning between postnatal days 20 and 24, mice were housed in groups on a 12-hour light-dark cycle with open access to food and water. All mouse handling and procedures were conducted as approved by the Stanford University Administrative Panel on Laboratory Animal Care.

### Tamoxifen Injections.

To prepare tamoxifen for injection, 1g of tamoxifen (Sigma, Cat # T5648–1G) was mixed with 10 ml of 200 proof ethyl alcohol (Gold Shield) at room temperature for 15 minutes while protected from light with foil. This mixture was then combined with 90 ml of corn oil (Sigma, Cat# C8267) and agitated at 37°C for 1–2 hours until fully dissolved, while continuing to protect from light. Aliquots of 1 ml were stored at −20°C. On the day of injections, tamoxifen aliquots (10mg/ml) were thawed to room temperature. Tamoxifen solution was injected with an insulin syringe intraperitoneally/subcutaneously with a series of two 30 μl injections at P10 and P11 or one 20 μl injection subcutaneously in the neck at P1.

### Immunoblotting.

Mice were anesthetized with isoflurane and then decapitated. Hippocampus and cortex were collected in RIPA buffer (50mM Tris-HCl, 150 mM NaCl, 1% Triton X-100, 0.1% SDS, 1 mM EDTA) with cOmplete, EDTA-free protease inhibitor cocktail (Roche, Cat# 11873580001) on ice. Tissue samples were homogenized with a dounce tissue grinder on ice, rotated at 4°C for 30 min, and finally centrifuged at 14,000 rpm at 4°C for 20 min. Supernatant was collected from the samples and protein content was quantified using a BCA assay (Thermo, Cat# 23225). Protein lysates were stored at −80°C until use.

Quantified protein lysates were added to Laemmli sample buffer with DTT. Samples were boiled at 95°C for 5 min, except in the case of immunoblotting for multi-pass transmembrane proteins, for which samples were not heated. Samples were run on 4–20% Criterion TGX precast gels (Bio-Rad, Cat# 5671094 & 5671095) to separate proteins by molecular weight. Proteins were transferred from gels to 0.2 μm nitrocellulose membranes (Bio-Rad, Cat# 1620112) using the Trans-blot turbo system (Bio-Rad) for 7 min at 25V. For detection of proteins, membranes were first blocked in 5% non-fat milk (Carnation) in TBST for one hour at room temperature, followed by incubation with primary antibodies diluted in 5% non-fat milk (Carnation) in TBST overnight at 4°C Membranes were washed with TBST three times for 10 min per wash prior to incubation with LI-COR secondary antibodies diluted in 5% non-fat milk (Carnation) in TBST at a concentration of 1:10,000. Membranes were subsequently imaged with the Odyssey CLx imaging system (LI-COR) with quantification carried out in Image Studio Lite 5.2. Protein quantifications were normalized to beta-actin and to protein levels of controls, as described in applicable figure legends.

### Immunohistochemistry.

Mice were anesthetized with isoflurane and then pericardially perfused with phosphate buffered saline (PBS) for 1 min followed by ice cold 4% paraformaldehyde (PFA) for 7 min at a rate of 1 μl/min. PBS and PFA were filtered through a 0.2 μm filter prior to perfusion. Brains were extracted and post-fixed in 4% PFA overnight at 4°C. PFA was removed, followed by thee washes with PBS. Brains were then placed in 30% sucrose (dissolved in PBS) for 24–48 hours and subsequently frozen in cryomolds (Tissue-Tek) in O.C.T. compound (Tissue-Tek). Brains were then sectioned coronally at 35 μm on a CM3050-S cryostat (Leica). To stain, free floating sections were first blocked for 1 hour at room temperature in blocking buffer (5% goat serum, 0.3% Triton X-100) and then incubated with primary antibodies diluted in blocking buffer overnight at 4°C. The next day, sections were washed three times for 15 min per wash in PBS at room temperature. Free floating sections were then incubated in secondary antibodies diluted at 1:1000 in blocking buffer for 2 hours at room temperature. Sections were washed three times for 15 min per wash in PBS at room temperature and then mounted on Superfrost Plus microscope slides (Fisherbrand) in 10% PBS. Once the sections were dry, slides were dipped in water and allowed to dry again. Coverslips (#1.5, VWR) were affixed with DAPI Fluoromount-G (Southern Biotech). Images of the hippocampus and primary visual cortex were taken with a Nikon confocal microscope using either the 20X air objective or 60X oil objective. Imaging conditions were consistent between control and Astro-NL123 cKO animals. Confocal images were analyzed, while blinded, using NIS-Elements Analysis Software (Nikon).

### Slice Electrophysiology.

Acute coronal brain slices (300 μm) containing the dorsal hippocampus were prepared from P45–50 Nlgn123 astrocyte conditional knockout mice and littermate controls, both of which had been injected with tamoxifen at P1. Mice were anesthetized with isoflurane and decapitated. The brain was rapidly removed and placed into oxygenated, ice cold cutting solution containing the following (in mM): 228 sucrose, 2.5 KCl, 1 NaH2PO4, 26 NaHCO3, 11 glucose,7 MgSO4–7H2O, and 0.5 CaCl2. Slices were recovered at 32°C for 30 minutes in oxygenated artificial cerebrospinal fluid (aCSF) containing the following (in mM): 119 NaCl, 2.5 KCl, 1 NaH2PO4, 26 NaHCO3, 11 glucose, 2.5 CaCl2 and 1.3 MgCl2 in ddH2O adjusted to a final osmolarity of 300 mOsm. Slices were then recovered at RT in oxygenated ACSF for at least another 30 minutes. Whole-cell voltage clamp recordings were performed on CA1 pyramidal neurons with the cells clamped at −70 mV and perfused continuously with room temperature, oxygenated ACSF at 1–2 ml / minute. Electrical signals were recorded at 5 kHz with a two channel MultiClamp 700B amplifier (Axon Instruments), digitalized with a Digidata 1440 digitizer (Molecular devices) that was in turn controlled by Clampex 10.7 (Molecular Devices). Recording pipettes were pulled from thin-walled borosilicate glass pipettes to resistances of 3–5 MΩ. mEPSCs were recorded with an internal solution containing (in mM): 135 cesium methanesulfonate, 8 NaCl, 10 HEPES, 2 Mg-ATP, 0.2 Na2-GTP, 0.1 spermine, 7 phosphocreatine, and 0.3 EGTA with a pH of 7.3 and adjusted to a final osmolarity of 300 mOsm. mIPSCs were recorded with an internal solution containing (in mM): 140 mM CsCl, 2 mM MgCl2, 5 mM EGTA, 10 mM Hepes, 0.3 mM Na3-GTP, and 4 mM Na2-ATP at pH 7.35 and adjusted to a final osmolarity of 300 mOsm. For mIPSC recordings, the calcium levels were reduced to 1 mM owing to the extremely high frequency observed in CA1 pyramidal neurons. mEPSCs were recorded in the presence of 0.5 μM tetrodotoxin (TTX; Cayman Chemical, Cat. 14964) and 50 μM picrotoxin (Tocris, Cat. 1128). mIPSCs were recording in the presence of 10 μM CNQX (Tocris, Cat. 1045), 50 μM D-AP5 (Tocris, Cat. 0106), and 0.5 μM TTX. Miniature events were analyzed blindly using Clampfit 10.7 via a template search and selected manually based on trained templates. Rise time and decay kinetics were calculated automatically as 10%−90% and 90%−10% peak amplitude of currents. Cumulative plots were generated based on the first 50 events from each recordings session that was either 5 minutes long or had at least 300 events.

### Plasmids.

To assess astrocyte morphology, the following plasmids were used: pAAV-GfaABC1D-Lck-mVenus and the helper plasmids pHelper and pAA5. The pAAV-GfaABC1D-Lck-mVenus construct was generated using PCR stitching to include the GfaABC1D promoter and a membrane targeting domain as described previously (Chai et al., 2017) and fused with mVenus in order to mark astrocyte membranes for analysis of astrocyte morphology.

### AAV Preparation.

For production of AAV (serotype AA5), HEK293T cells were co-transfected using the calcium phosphate method with pAAV-GfaABC1D-Lck-mVenus and the helper plasmids (pHelper and pAA5) at 4 μg per plasmid per 30 cm^2^ culture area. Approximately 12 hours after transfection, media was changed. At, ~72 hours after the transfection, the HEK293T cells were collected using PBS with 10 mM EDTA and then spun down at 1500 × g. The cell pellets were resuspended in freezing buffer and subjected to 3 freeze-thaw cycles, alternating between 37C and a dry ice bath. Lysates were then incubated with 50 units / ml of benzonase nuclease at 37C for 30 minutes followed by a spin at 3000 × g for 30 minutes. The supernatant was loaded into discontinuous iodixanol gradient and centrifuged at 65,000 rpm for 3 hours. The 40% iodixanol fraction was collected and concentrated using 100,000 MWCO centricon columns. Filtrate was washed with several charges of MEM and then aliquoted and stored at −80C until use. Although viral titer was not determined, purified AAV was injected in vivo at various dilutions in order to identify a dilution that allowed sparse targeting of astrocytes for morphological reconstruction; thus, final viral titer was relatively low.

### Stereotactic Injections.

Prior to surgery, P21 mice were anesthetized with tribromoethanol (Avertin) at a dosage of 125–300 mg/kg and Buprenorphine Sustained Release was administered at 0.5 mg/kg to provide analgesia. Mouse heads were shaved and then cleaned with Betadine prior to incision of the scalp. Using a stereotactic rig (Kopf) for targeting, AAV expressing membrane-targeted mVenus under the control of the GFAP promoter was injected into CA1 (coordinates from bregma: A-P −1.8, M-L ±1.15, D-V −1.4) or V1 (coordinates from bregma: A-P −3.99, M-L ±2.6, D-V −1.5). Virus was injected through a glass pipette attached to a syringe pump (SP101i, World Precision Instruments) at a speed of 0.15 μl/min at a volume that was empirically determined to sparsely infect astrocytes (0.5 μl). Following injection, the incision was closed with 4–0 nylon sutures (Unify). Mice were allowed to recover in a clean cage on a heating pad prior to transfer to a clean home cage. Sutures were removed 10 days post-surgery.

### Astrocyte Morphology.

Mice were stereotactically injected with AAV expressing membrane-targeted mVenus under the control of the GFAP promoter at P21 and then perfused, as described in the immunohistochemistry section, at P35. Brains were post-fixed in 4% PFA for 24 hours at 4°C. Brains were then washed 3 times in PBS and then vibratome sectioned at 100μm in PBS. Sections were counterstained with DAPI (D8417, Sigma) and mounted on gelatin-coated slides (FD Neurotechenologies, Cat# PO101) in 10% PBS. Once sections were dry, slides were dipped in water and again allowed to dry. Coverslips (#1.5, VWR) were affixed with Fluoromount-G (Southern Biotech). Images were taken on a Zeiss AiryScan microscope using the same imaging parameters for controls and Astro-Nlgn123 cKO animal. While blinded, astrocyte morphologies were reconstructed with 3D rendering in Imaris and astrocyte volumes were measured.

### Data Analysis and Statistics.

For electrophysiology experiments, data were analyzed with Clampfit 10.7 (Molecular Devices). For immunoblot, immunohistochemistry, and astrocyte morphology experiments, unpaired two-tailed t-tests were used to assess statistical significance. For electrophysiology experiments, unpaired two-tailed t-tests were used to analyze data plotted in bar graphs (e.g., rise time) and Kolmogorov-Smirnov tests were used to assess statistical significance of cumulative curves. For bar graphs, data are depicted as means ± SEM. For all experiments, significance is indicated by * p<0.05, **p<0.01 or ****p<0.0001.

## Supplementary Material

Supplement 1

## Figures and Tables

**Key resources table:** 

Reagent type (species) or resource	Designation	Source or reference	Identifiers	Additional information
Strain, strain background (Mus musculus)	Aldh1l1-Cre/ERT2 BAC transgenic mice	The Jackson Laboratory	RRID:IMSR_JAX:029655	
Strain, strain background (Mus musculus)	Nlgn123 cKO mice	Südhof lab		
Cell line	HEK293T cells	ATCC	RRID: CVCL_0063	
Antibody	Anti-Nlgn1	Synaptic Systems	129111, RRID:AB_887747	1:2000
Antibody	Anti-Nlgn2	Synaptic Systems	129203, RRID:AB_993014	1:1000
Antibody	Anti-Nlgn3	Synaptic Systems	129311, RRID:AB_2151947	1:2000
Antibody	Anti-β Actin	Sigma Aldrich	A1978, RRID:AB_476692	1:5000
Antibody	Anti-VGluT2	Millipore Sigma	AB2251, RRID:AB_1587626	1:1000
Antibody	Anti-VGluT1	TCS	YZ6089, RRID:AB_2861224	1:1000
Antibody	Anti-Synaptotagmin 1	TCS	V216	1:1000
Antibody	Anti-Synaptobrevin 2	TCS	P939	1:1000
Antibody	Anti-SNAP25	TCS	P913, RRID:AB_2861227	1:1000
Antibody	Anti-PSD95	Synaptic Systems	124011, RRID:AB_10804286	1:1000
Antibody	Anti-Mint1	TCS	P730	1:1000
Antibody	Anti-GRIP	BD Transduction Laboratories	611319, RRID:AB_398845	1:1000
Antibody	Anti-GluR2	Neuromab	75-002, RRID:AB_2232661	1:1000
Antibody	Anti-GluR1	Neuromab	75-327, RRID:AB_2315840	1:1000
Antibody	Anti-Gephyrin	Neuromab	75-444, RRID:AB_2636852	1:1000
Antibody	Anti-CASK	Neuromab	75-000, RRID:AB_2068730	1:1000
Antibody	Anti-Calbindin	Sigma	C9848, RRID:AB_476894	1:2000
Antibody	Anti-Mouse SPARC-like1	R&D Systems	BAF2836, RRID:AB_2195096	0.5 μg/ml
Antibody	Anti-SPARC	DSHB	mAB 236, RRID:AB_2617208	1 μg/ml
Antibody	Anti-Myelin Basic Protein	EnCOR Biotechnology	CPCA-MBP, RRID:AB_2572352	1:5000
Antibody	Anti-Kir4.1	Millipore Sigma	AB5818, RRID:AB_92053	1:1000
Antibody	Anti-EEAT1	Abcam	ab416, RRID:AB_304334	1:1000
Antibody	Anti-MAP2	EnCOR Biotechnology	CPCA-MAP2, RRID:AB_2138173	1:500
Antibody	Anti-Gephyrin	Synaptic Systems	147318, RRID:AB_2661777	1:200
Antibody	Anti-GAD67	Millipore	MAB5406, RRID:AB_2278725	1:1000
Antibody	Anti-VGluT1	Millipore	AB5905, RRID:AB_2301751	1:1000
Antibody	Anti-MAP2	Sigma Aldrich	M1406, RRID:AB_477171	1:1000
Antibody	Anti-Homer1	TCS	YZ6081	1:1000
Antibody	Anti-NeuN	Millipore Sigma	MAB377, RRID:AB_2298772	1:1000
Antibody	Anti-Sl00	Abcam	ab868, RRID:AB_306716)	1:1000
Antibody	Alexa fluor 647, goat anti rabbit IgG	Invitrogen	A-21245, RRID:AB_141775)	1:1000
Antibody	Alexa fluor 546, goat anti rabbit IgG	Invitrogen	A-11035, RRID:AB_143051)	1:1000
Antibody	Alexa fluor 633, goat anti- mouse IgG	Invitrogen	A-21052, RRID:AB_2535719	1:1000
Antibody	Alexa fluor 488, goat anti guinea pig IgG	Invitrogen	A11073, RRID:AB_2534117	1:1000
Antibody	Alexa fluor 647, goat anti chicken IgG	Invitrogen	A21449, RRID:AB_1500594	1:1000
Antibody	Alexa fluor 546, goat anti guinea pig IgG	Invitrogen	A-11074, RRID:AB_2534118	1:1000
Antibody	Alexa fluor 488, goat anti mouse IgG	Invitrogen	A-11001, RRID:AB_2534069	1:1000
Antibody	IRDye 800CW donkey anti chicken	Licor	926-32218, RRID:AB_1850023	1:10000
Antibody	IRDye 800CW donkey anti goat	Licor	926-32214, RRID:AB_621846	1:10000
Antibody	IRDye 680RD donkey anti guinea pig	Licor	926-68077, RRID:AB_10956079	1:10000
Antibody	IRDye 800CW donkey anti rabbit	Licor	926–32213, RRID: AB_621848	1:10000
Antibody	IRDye 680LT donkey anti mouse	Licor	926–68022, RRID: AB_621848	1:10000
Antibody	IRDye 800CW donkey anti mouse	Licor	926–32212, RRID: AB_10715072	1:10000
Chemical compound, drug	Picrotoxin	Tocris	1128	
Chemical compound, drug	CNQX	Tocris	1045	
Chemical compound, drug	D-AP5	Tocris	0106	
Chemical compound, drug	Tetrodotoxin	Cayman Chemical	14964	
Chemical compound, drug	Tamoxifen	Sigma	T5648-1G	
Chemical compound, drug	Corn Oil	Sigma	C8267	
Software, algorithm	Clampfit	Molecular Devices	N/A	
Software, algorithm	pClamp	Molecular Devices	RRID:SCR_011323	
Software, algorithm	Prism	Graphpad software inc	RRID: SCR_002798	
Software, algorithm	Image Studio Lite	Licor	RRID: SCR_014211	
Software, algorithm	Imaris	Oxford Instruments	RRID:SCR_007370	
Software, algorithm	NIS-Elements Basic Research	Nikon	RRID:SCR_002776	
