# Supplementary material for: Deletion of Neuroligins from Astrocytes Does Not Detectably Alter Synapse Numbers or Astrocyte Cytoarchitecture by Maturity
Source: bioRxiv. 2025 Jan 6:2023.04.10.536254. Originally published 2023 Apr 10. Preprint. [Version 2] doi: 10.1101/2023.04.10.536254 (PMC10120619; doi:10.1101/2023.04.10.536254)
Supplement: Supplementary file 1 [file NIHPP2023.04.10.536254v2-supplement-1.pdf]

bioRxiv preprint doi: https://doi.org/10.1101/2023.04.10.536254; this version posted January 6, 2025. The copyright holder for this preprint (which was not certified by peer review) is the author/funder, who has granted bioRxiv a license to display the preprint in perpetuity. It is made available under aCC-BY 4.0 International license.

SUPPLEMENTARY FIGURES and FIGURE LEGENDS

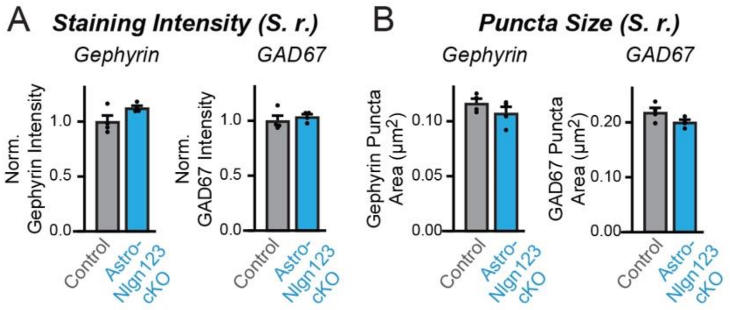

Figure S1: **Conditional deletion of astrocytic *Nlgn1-3* at P1 does not alter the number or size of inhibitory synapses in the CA1-region *Stratum Radiatum***

(**A**) Quantification of total Gephyrin (left) or GAD67 (right) immunofluorescence in CA1 *Str. Radiatum* from astrocyte Nlgn1-3 cKO and littermate control mice. Images of hippocampal sections were taken at 60X magnification. Total immunofluorescent signal was first internally normalized to MAP2 and then to average gephyrin (left) or GAD67 (right) immunofluorescence level in control mice.

(**B**) Quantification of puncta density for Gephyrin (left) and GAD67 (right) CA1 *Str. Radiatum* from astrocyte Nlgn1-3 cKO and littermate control mice.

Representative images are shown in Fig. 4G. Data are means ± SEM with statistical significance determined by unpaired two-tailed t-test (n=4, 2 male & 2 female).

bioRxiv preprint doi: https://doi.org/10.1101/2023.04.10.536254; this version posted January 6, 2025. The copyright holder for this preprint (which was not certified by peer review) is the author/funder, who has granted bioRxiv a license to display the preprint in perpetuity. It is made available under aCC-BY 4.0 International license.

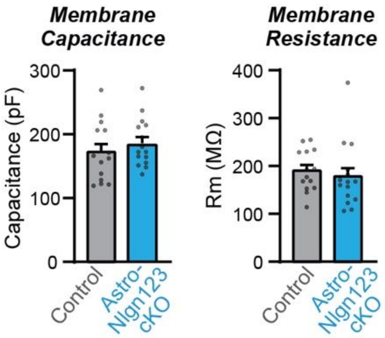

Figure S2: **Conditional deletion of astrocytic *Nlgn1-3* at P1 does not alter CA1 pyramidal neuron membrane properties**

(**A**) Summary graph of membrane capacitance from CA1 pyramidal neurons in acute slices from astrocyte Nlgn1-3 cKO and littermate controls injected with tamoxifen at P1 and recorded at P44 – P50.

(**B**) Same as (**A**) but for membrane resistance.

Data are means ± SEM with statistical significance determined by unpaired two-tailed t-test (n = 14-15 cells / 3 mice per genotype).

bioRxiv preprint doi: https://doi.org/10.1101/2023.04.10.536254; this version posted January 6, 2025. The copyright holder for this preprint (which was not certified by peer review) is the author/funder, who has granted bioRxiv a license to display the preprint in perpetuity. It is made available under aCC-BY 4.0 International license.

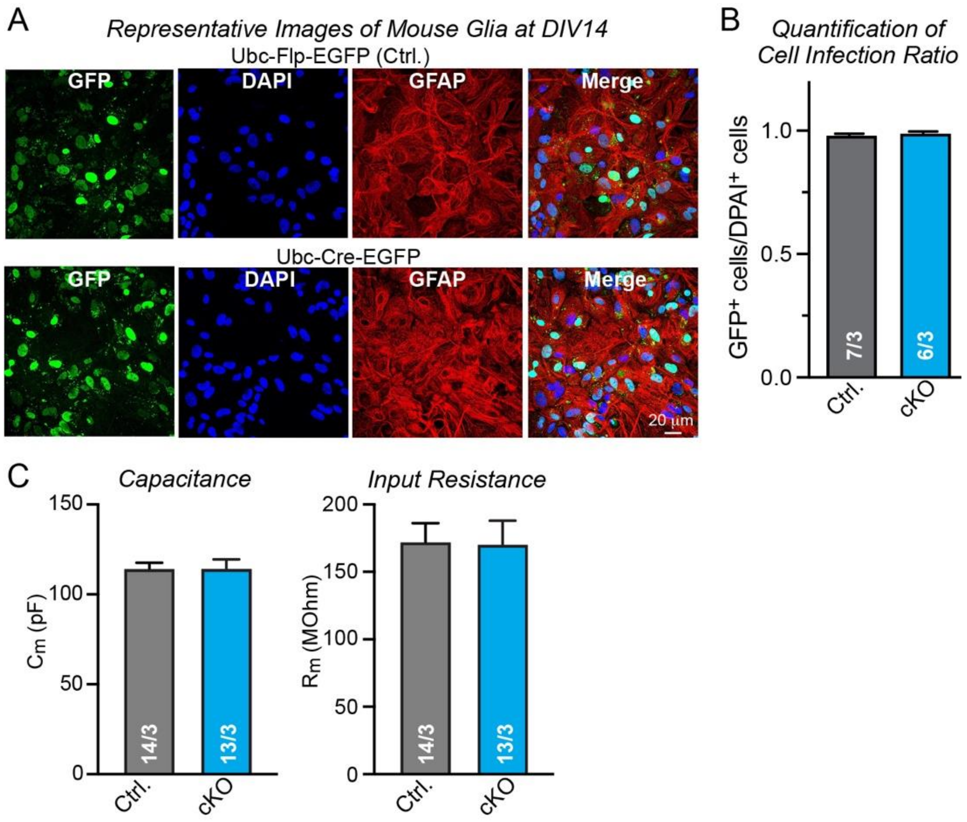

Figure S3: **Further data characterizing the effect of a deletion of glial *Nlgn1-3* on co-cultured human neurons**

(**A**) Representative images of primary mouse glia cultures from *Nlgn1-4* quadruple cKO mice infected with lentiviruses encoding FLP-EGFP (Ctrl.) or Cre-EGFP fusion proteins and stained for GFP, DAPI, and GFAP as glial marker. Note that the levels of EGFP expression differ among glia cells but that based on qRT-PCR measurements the lower levels of Cre-EGFP in some glial cells are sufficient for complete recombination of *Nlgn1*, *Nlgn2*, and *Nlgn3* genes (*Nlgn4* is constitutively deleted).

(**B**) Summary graph of the ratio of EGFP-expressing to DAPI-stained cells demonstrates that nearly all cells in the culture are infected by the lentiviruses.

(**C**) Summary graphs of the membrane capacitance (left) and input resistance (right) monitored in human neurons that are co-cultured with mouse glia expressing or lacking all neuroligins (Nlgn1-4).

Data are means ± SEM with statistical significance determined by unpaired two-tailed t-test (n = 14-15 cells / 3 mice per genotype).
