# Supplementary material for: Astrocytic Neuroligins Are Not Required for Synapse Formation or a Normal Astrocyte Cytoarchitecture
Source: bioRxiv. 2023 Apr 10:2023.04.10.536254. Preprint. [Version 1] doi: 10.1101/2023.04.10.536254 (PMC10120619; doi:10.1101/2023.04.10.536254)
Supplement: Supplementary file 1 [file NIHPP2023.04.10.536254v1-supplement-1.pdf]

bioRxiv preprint doi: https://doi.org/10.1101/2023.04.10.536254; this version posted April 12, 2023. The copyright holder for this preprint (which was not certified by peer review) is the author/funder, who has granted bioRxiv a license to display the preprint in perpetuity. It is made available under aCC-BY 4.0 International license.

SUPPLEMENTARY FIGURES and FIGURE LEGENDS

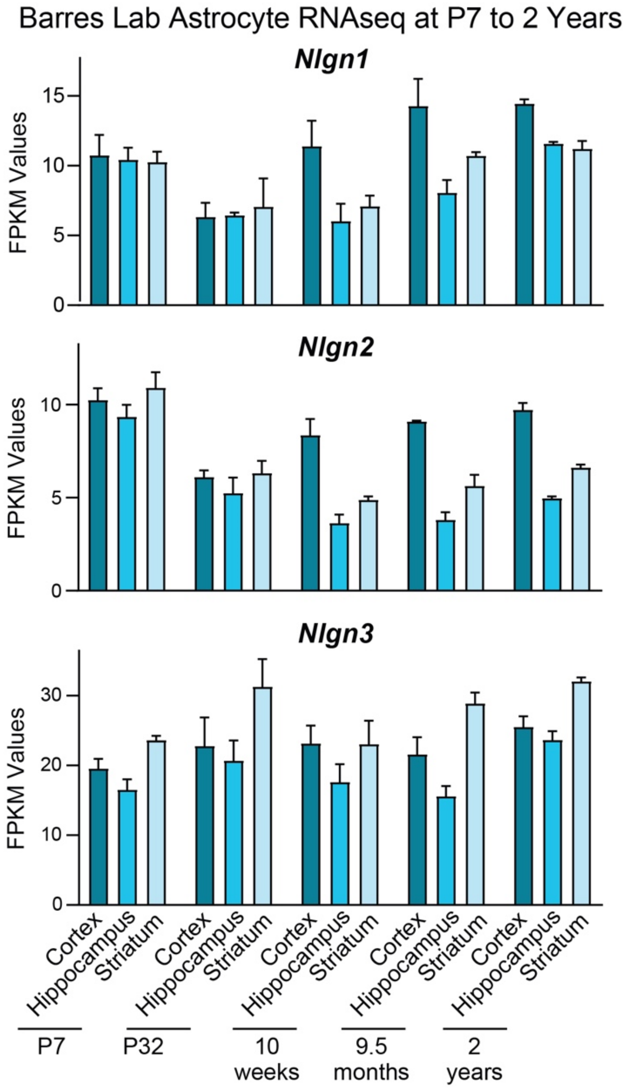

Figure S1: **Expression of *Nlgn1-3* by aging astrocytes of the cortex, hippocampus, and striatum as analyzed by the pioneering Barres lab datasets**

Comparison of mRNA expression levels for Neuroligins 1-3 by astrocytes in the cortex, hippocampus, and striatum across five developmental timepoints, as published by the Barres lab (Clarke et al., 2018, www.brainrnaseq.org). Astrocytic transcripts were purified using translating ribosome affinity purification (TRAP) from *Aldh1l1-eGFP-L10a* mice.

bioRxiv preprint doi: https://doi.org/10.1101/2023.04.10.536254; this version posted April 12, 2023. The copyright holder for this preprint (which was not certified by peer review) is the author/funder, who has granted bioRxiv a license to display the preprint in perpetuity. It is made available under aCC-BY 4.0 International license.

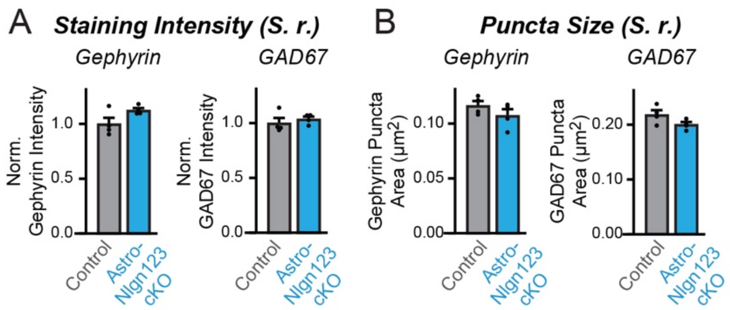

<u>Figure S2</u>: **Conditional deletion of astrocytic *Nlgn1-3* at P1 does not alter the number or size of inhibitory synapses in the CA1-region *Stratum Radiatum***

(**A**) Quantification of total Gephyrin (left) or GAD67 (right) immunofluorescence in CA1 *Str. Radiatum* from astrocyte Nlgn1-3 cKO and littermate control mice. Images of hippocampal sections were taken at 60X magnification. Total immunofluorescent signal was first internally normalized to MAP2 and then to average gephyrin (left) or GAD67 (right) immunofluorescence level in control mice.

(**B**) Quantification of puncta density for Gephyrin (left) and GAD67 (right) CA1 *Str. Radiatum* from astrocyte Nlgn1-3 cKO and littermate control mice.

Representative images are shown in Fig. 4G. Data are means ± SEM with statistical significance determined by unpaired two-tailed t-test (n=4, 2 male & 2 female).

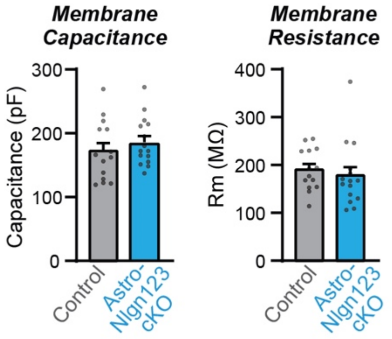

<u>Figure S3</u>: **Conditional deletion of astrocytic *Nlgn1-3* at P1 does not alter CA1 pyramidal neuron membrane properties**

bioRxiv preprint doi: https://doi.org/10.1101/2023.04.10.536254; this version posted April 12, 2023. The copyright holder for this preprint (which was not certified by peer review) is the author/funder, who has granted bioRxiv a license to display the preprint in perpetuity. It is made available under aCC-BY 4.0 International license.

(**A**) Summary graph of membrane capacitance from CA1 pyramidal neurons in acute slices from astrocyte Nlgn1-3 cKO and littermate controls injected with tamoxifen at P1 and recorded at P44 – P50.

(**B**) Same as (**A**) but for membrane resistance.

Data are means ± SEM with statistical significance determined by unpaired two-tailed t-test (n = 14-15 cells / 3 mice per genotype).
